# HuangE Capsules Improve Bladder Function in BOO-induced Overactive Bladder Rats: Network Pharmacology and Experimental Validation

**DOI:** 10.2174/0113862073373430250415070358

**Published:** 2025-04-15

**Authors:** Peizhe Li, Yuewen Pang, Shiyu Zhao, Heyang Liu, Siyu Han, Ran Zhong, Shuang He, Jing Shi, Haisheng Cheng, Huijie Gong, Yongji Yan, Junyao Duan

**Affiliations:** 1 Department of Urology, Dongzhimen Hospital, Beijing University of Chinese Medicine, Beijing, China

**Keywords:** Overactive bladder, urology, bladder outlet obstruction, HuangE capsules, bladder function, BOO-induced, network pharmacology, ROCK1, ROCK2

## Abstract

**Aims:**

Our objective is to assess the therapeutic impact of HEC on OAB rats and investigate potential mechanisms.

**Background:**

Overactive bladder (OAB) is a syndrome of urinary storage symptoms characterized by “urinary urgency with or without urinary acute incontinence, usually accompanied by increased daytime and nocturnal urination”, which impacts patients’ quality of life. We found the potential therapeutic impact of HuangE capsules (HEC) on OAB patients through clinical practice. However, the exact effect and mechanism of action remain unclear.

**Methods:**

We developed a "drugs- active ingredients- targets- diseases" network and employed the pathway enrichment analysis to identify the potential mechanisms of HEC on OAB. Bladder outlet obstruction (BOO) models and sham-operated ones were established in healthy male Wistar rats through surgical procedures. Following 28 days of continuous gavage administration of HEC, saturated copper sulfate test paper was utilized to quantify the frequency of urination over a 24-hour period. Subsequently, cystostomy was conducted to perform cystometry, and Masson staining was applied to a portion of the bladder tissue. Finally, we investigated the Rho/Rho-kinase pathway's expression and assessed the oxidative stress and inflammatory factor levels in the rat bladder through western blotting and ELISA techniques.

**Results:**

Through network pharmacological analysis, we identified RhoA/Rho-kinase pathway and cytokine including TNF-α, IL-6, SOD and MDA as potential mechanisms of HEC on OAB. The rats in the 2× HuangE group exhibited significantly enhanced urodynamic outcomes and decreased 24-hour urination frequency compared to the model group. Masson staining indicated a decrease in the proportion of collagenous tissue and an improvement in histomorphology. We observed a decrease expression of RhoA, ROCK1, and ROCK2 protein in the bladder tissue of 2× HuangE group rats, along with elevated SOD levels and decreased levels of TNF-α, IL-6, and MDA.

**Discussion:**

Mechanistically, HEC suppressed the Rho/Rho-kinase pathway in bladder tissue, reducing the expression of RhoA, ROCK1, and ROCK2, while decreasing inflammatory cytokines and oxidative stress, thereby alleviating fibrosis and improving urodynamics. Given that HEC also inhibits prostatic proliferation, a synergistic benefit for OAB patients with BPH is plausible. Gender differences and efficacy in neurogenic OAB warrant further investigation. Overall, HEC is a promising multi-target TCM option for OAB, though clinical validation remains necessary.

**Conclusion:**

HEC could improve bladder function and morphology in BOO-induced OAB rats by reducing the expression of RhoA, ROCK1, and ROCK2 and lowering levels of oxidative stress and inflammation.

## 1 INTRODUCTION

Overactive bladder (OAB) was defined by the International Continence Society in 2002 as a syndrome of urinary storage symptoms characterized by "urinary urgency with or without urinary acute incontinence, usually accompanied by increased daytime and nocturnal urination”, significantly impacting the patients’ quality of life (QoL) [1, 2]. In the United States, around 14.5 % of men are afflicted with OAB, and its overall prevalence has notably risen over the past two decades [3]. While OAB may pose challenges for complete cure, effective management can assist in symptom alleviation and enhancing patients' QoL. Antimuscarinic agents, such as tolterodine, could diminish bladder smooth muscle contractions and serve as the primary pharmacologic option for OAB. Nonetheless, they may lead to side effects like dry mouth, constipation, acute urinary retention, or visual impairment, complicating their long-term utilization [4].

Exploring novel treatment approaches for OAB from natural remedies could be advantageous. In traditional Chinese medicine (TCM) theory, “urinary urgency” and “urinary frequency” are commonly associated with “retention of urine” (TCM name: *Long Bi*) or “stranguria” (TCM name: *Lin Zheng*), primarily caused by “Dampness-Heat (TCM name: *Shi Re*)”. The principle of “cleaning heat and disinhibit dampness (CHDD, TCM name: *Qing Re Li Shi*)” should be applied when using TCM herbal medicine, which is a recognized and recommended TCM therapy on OAB management [5]. CHDD is closely associated with the anti-inflammatory and antioxidant effects recognized in modern medicine. HuangE Capsules (HEC) is a traditional Chinese compound preparation that embodies the CHDD principle [6], comprising a blend of twelve traditional herbs (composition detailed in Table **1**). It has successfully cleared the safety assessment of China National Medical Products Administration (CNMPA) and is authorized for commercial distribution (approval number: Z20110006). HEC has been commonly used in the retention of urine management of TCM, and its pharmacological mechanism in treating benign prostatic hyperplasia (BPH), a subtype of retention of urine, has been demonstrated. In clinical practice, bladder function improvements were observed in BPH patients following HEC administration, suggesting a potential therapeutic effect on OAB [7].

We explored the potential targets of HEC in the treatment of diseases using the Traditional Chinese Medicine Systems Pharmacology Database and Analysis Platform (TCMSP) and established a “drugs-active ingredients-targets-diseases” network by analyzing interactions between predicted HEC targets and known OAB disease targets. Gene Ontology (GO) and Kyoto Encyclopedia of Genes and Genomes (KEGG) pathway enrichment analysis, along with molecular docking, were employed to identify active mechanisms. Subsequently, we conducted experimental validation on the OAB rat model. The results indicate that HEC could improve bladder function in BOO-induced OAB rats by reducing the expression of the Rho/Rho-kinase pathway and lowering levels of oxidative stress and inflammation. This study offers a novel perspective and scientific foundation for TCM treatment strategies of OAB.

## 2 MATERIALS AND METHODS

### 2.1 Acquisition of Active Ingredients and Protein Target in HEC

The active components of HEC underwent screening using the TCMSP. The criteria set included oral bioavailability ≥ 30 % and a drug-like index ≥ 0.18. Gene symbols and names were retrieved from the PubChem (https://pubchem.ncbi.nlm.nih.gov/) and SwissTargetPrediction (www.swisstargetprediction.ch) databases. The targets of HEC were ultimately identified through the consolidation and removal of duplicates.

### 2.2 Acquisition of Therapeutic Genes on OAB

We utilized the GeneCards (www.genecards.org), DrugBank (www.drugbank.com), and PharmGKB (www.pharmgkb.org) databases to obtain target genes by searching for “overactive bladder”. They were finally identified after merging and eliminating duplicates. The intersecting targets of HEC and OAB were determined using the Venn package in the R programming language.

### 2.3 Target Prediction by Protein–protein Interaction Network Analysis

Cytoscape version 3.9.1 was employed to analyze and visualize the topological properties of the exported data and to construct a network representing the relationship of “drugs-active ingredients-targets-diseases” Subsequently, the intersecting targets were imported into the String database (https://cn.string-db.org/) with the species specified as "Homo sapiens" to obtain the protein–protein interaction (PPI) network. The minimum action score was set above 0.7, and isolated protein nodes were hidden.

### 2.4 GO Function and KEGG Pathway Enrichment Analysis

We performed GO function and KEGG pathway analysis of core targets using the DAVID database [8]. These analyses are employed to enrich potential targets by gene, chemical, and protein information. Heatmap was plotted by https://www.bioinformatics.com.cn (last accessed on 20 June 2024), an online platform for data analysis and visualization [9].

### 2.5 Animals

Healthy specific-pathogen-free Wistar rats (male, 7 weeks old, 330 ± 20 g) were obtained from Beijing Vital River Laboratory Animal Technology Co., Ltd. The rats were housed at room temperature with a relative humidity of 55 ± 5 %. Throughout this period, the animals had unrestricted access to water and standard chow. All animal experiments were approved by the Ethics Committee for Animal Experiments of Beijing University of Chinese Medicine (Approval ID: BUCM- 2023082811- 3136). Animal care and surgical procedures adhered strictly to the National Laboratory Animal Guidelines.

### 2.6 Bladder Outlet Obstruction Model

All rats (n = 40) were randomly divided into five groups: Sham (sham-operated), BOO, 0.5× HuangE (half of normal dosage), 1× HuangE (normal dosage of HEC), and 2× HuangE (double of normal dosage). The BOO models were established based on previous literature [10]. Anesthesia was induced by administering titetamme/zolazepam (Virbac S.A., France) 50 mg/kg *via* intraperitoneal injection [11]. Subsequently, a 3 cm longitudinal incision was made in the midline of the suprapubic bone upon confirmation of anesthesia. The bladder neck and proximal urethra are detached carefully after locating the bladder. A calibrating rod (diameter = 1 mm) was positioned parallel to the proximal urethra and withdrawn after ligation with a 3- 0 non-absorbable silk thread. Amoxicillin hydrochloride injection (150 mg/kg) was administered to prevent infection. The Sham group did not undergo ligation; however, all other surgical operations and steps were consistent with those in the BOO model (Fig. **1A**). All procedures were performed by the same operator.

### 2.7 Administration of HEC

HEC was obtained from Zhejiang Conba Pharmaceutical Co., Ltd. It is a compound preparation that is authorized for marketing by CNMPA and protected by a patent covering its drug formulation and manufacturing process. Since the amount of heat dissipated per unit of body surface area is similar among species, it is reasonable to use body surface area-based dose conversion to determine the appropriate drug dosage for experimental animals [12]. The recommended dosage of HEC for an adult male (weighing approximately 70 kg) is 1.6 g, three times daily. Given the human-to-rat equivalent dose ratio of 6.3, the daily dosage of HEC per rat can be calculated as follows: 6.3× 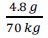 × 0.33 kg= 0.14256 g. Accounting for potential conversion errors, the final equivalent dosage of HEC for rats was determined to be 0.16 g per rat per day.

The HEC powder, after capsule coat removal, was dissolved in pure water without the addition of any excipients to prepare suspensions. Rats in the 0.5× HuangE (0.04 g/mL) group, 1× HuangE (0.08 g/mL) group, and 2× HuangE (0.16 g/mL) group received 2 mL of the suspensions *via* gavage from day 3 to day 30 post-BOO surgery. Rats in the Sham and BOO groups were provided with 2 mL of water daily.

### 2.8 24-hour Urination Frequency Test

Counting the number of voids in rats enables the analysis of altered bladder function across different groups. The frequency of urination can be measured using copper sulfate supersaturated test strips, as urine forms distinct borders upon contact with the strips. On post-BOO surgery day 30, all rats were housed individually in metabolic cages. Prepared copper sulfate supersaturated test strips were placed beneath the cages to measure the number of urinations over a 24-hour period (Fig. **1B**), following the previously established method [13, 14]. The strips were replaced every 8 hours. Each imprint on the copper sulfate paper, with distinct edges, was counted as one voiding. Three test strips were collected, and the urination marks were counted and summed to determine the total number of urinations per rat over a 24-hour period.

### 2.9 Cystostomy

Given the physiological characteristics of male rats, an invasive cystostomy was necessary to conduct intravesical pressure measurements [15]. Immediately following the 24-hour urination test, all rats underwent cystostomy. While the rats were anesthetized, the abdominal cavity was opened. A polyethylene 50 (PE50) catheter was inserted into the bladder through its dome and tunneled subcutaneously to the back of the rat's neck (Fig. **1C**). After closing the abdominal cavity, an amoxicillin hydrochloride injection (150 mg/kg) was administered to prevent infection.

### 2.10 Continuous Cystometry in the Awake State

Continuous cystometry was performed on day 3 post-cystostomy while the rats were awake. The rats were immobilized on a special rack to facilitate the observation of voiding and urine collection with a posterior jugular catheter connected to a pressure transducer and an infusion pump *via* a three-way valve (Fig. **1C**). The micro-infusion pump continuously delivered saline into the bladder at a rate of 0.4 mL/min. Bladder pressure curves were plotted using the BL-420N Bio-signal Acquisition and Analysis System with accompanying software (Chengdu Techman Software Co., Ltd.). Experimental recording commenced after the rat completed its first urination. The start and end time points of each subsequent voiding event were recorded. At least three voiding cycles were recorded, during which the parameters: baseline pressure, peak pressure, minimum micturition threshold, and average micturition interval, were measured. A small measuring cup was used to collect urine and measure the volume of each voiding. The volume of infused saline between two voidings can be calculated by using the time difference between the start and end of each voiding event and a constant infusion rate of 0.4 mL/min. Residual urine volume was determined by sequentially subtracting the volume of urine from subsequent voidings.

### 2.11 Disposal for Rat Bladder Tissue

After the urodynamic examination, the rats were weighed, and their bladder tissues were obtained under anesthesia. The wet weight of each bladder was recorded to calculate the ratio of wet bladder to body weight. We clipped about 0.02 g of the fundus of bladder tissue from each sample and placed it into 10 % neutral formalin for 12 hours before embedding by paraffin. The remaining tissue was then homogenized and centrifuged, followed by the addition of RIPA cell lysate to extract tissue proteins for the following tests.

### 2.12 Histomorphological Analysis

Paraffin-embedded samples were cut into 5 µm slices and mounted on slides for Masson trichrome staining. The stained sections were scanned with a NanoZoomer^®^ S360 digital slide scanner and viewed using NDP.view 2.9.22 RUO software (Hamamatsu Photonics K.K.). We assessed the morphological changes in the bladder tissue and calculated the ratio of collagen tissue to all stained tissue by ImageJ software.

### 2.13 Enzyme-linked Immunosorbent Assay (ELISA)

Interleukin 6 (IL-6, serial number: H007-1-1), tumor necrosis factor alpha (TNF-α, serial number: H052-1-1), superoxide dismutase (SOD, serial number: A001-1-1) and malondialdehyde (MDA, serial number: A003-1-1) levels in samples between different groups were assessed using dedicated ELISA kits (Nanjing Jiancheng Biological Engineering Research Institute). The experiments were conducted following the guidelines provided by the manufacturer.

### 2.14 Western Blot (WB)

Protein quantification was conducted using the BCA Protein Assay Kit (Aoqing Biotechnology Ltd., Beijing, China). Target Proteins were separated by electrophoresis on 8 % and 12.5 % polyacrylamide gels according to their molecular weights. The separated proteins were then transferred onto polyvinylidene difluoride membranes. Blocking of the membranes was carried out using 5 % skimmed milk in Tris-buffered saline containing 0.1 % Tween^®^ 20. Subsequently, the membranes were probed with antibodies against RhoA (ProteinTech Group, China), ROCK1 (ProteinTech Group, China), ROCK2 (ProteinTech Group, China), and β-Actin (ProteinTech Group, China). After washing, the membranes were incubated with secondary antibodies at room temperature for one hour. Immunoreactive signals were detected using an ECL kit (Biorigin Inc., Beijing) and a luminescence imaging analyzer (Tanon Science & Technology Co., Ltd., China). Finally, band intensity was digitized with ImageJ software and normalized to that of β-actin.

### 2.15 Statistical Analysis

Statistical analysis was performed using SPSS version 29.0 software (IBM Corp., Armonk, NY, USA). One-way ANOVA was employed for pairwise comparisons between groups when data exhibited normal distribution. The least-significant difference (LSD) method was utilized for homogeneous variance and Dunnett's T3 method for non-homogeneous variance, with significance set at *P* < 0.05. Use GraphPad Prism software version 10.2.3 (Dotmatics Ltd., USA) for bar charting.

## 3 RESULTS

### 3.1 PPI Network and Potential Targets

A total of 646 targets associated with HEC and 265 ones related to OAB were obtained. The intersection of these datasets yielded 49 common targets, as depicted in Fig. (**2**) (more details can be found in Supplementary-Table **S1**). The network diagram illustrating "drugs-active ingredients-targets-pathways-diseases" delineates the connections among them. The potential drug components of HEC ranked by degree, indicated that the pharmacological effects of HEC may be attributed to these components (shown in Table **2**). Subsequently, the PPI network was constructed through the String database (shown in Fig. **2**), highlighting IL-6 and TNF proteins with the highest degree, situated centrally in the network, suggesting their potential role and close associations with other nodes in the mechanism of HEC. Given that the active ingredients and drug targets are derived from *in vitro* databases, the results of cyberpharmacology should be considered predictions rather than definitive outcomes. Consequently, experimental validation of our predictions will be necessary.

### 3.2 GO and KEGG Pathway Enrichment Analysis

The GO analysis encompassed biological processes (BP), cellular components (CC), and molecular functions (MF), from which the top ten pathways are shown in the Fig. (**3**). A. KEGG analysis enhances gene collections based on specific experimental conditions to determine the significance of these functions or pathways under those conditions [16]. The identified potential pathways of HEC include the G protein-coupled acetylcholine receptor signaling pathway, calcium channel signaling pathway, cAMP signaling pathway, and TNF pathway *et al* (shown in the Fig. **3**). Integrating insights from prior literature and the PPI network results, we propose that the muscarinic receptor-coupled Rho/Rho-kinase pathway and inflammatory factors like IL-6 and TNF-α could serve as potential pathways and targets for HEC's mechanism of action.

### 3.3 Urination Behaviors and Urodynamic Outcomes

Over a 24-hour period, rats in the BOO group exhibited significantly higher urination frequency than those in the Sham group (*p* < 0.001). While there was a reduction in the 1× and 2× HuangE groups compared to the BOO group (*p* < 0.01), the difference between these groups was not significant. Similarly, the urination frequency in the 0.5× group decreased, yet the variance compared to the BOO group was not statistically significant (shown in the Fig. **4A**).

Cystometry provided insights into the rats' bladder function. There were no significant differences in the baseline and peak pressures between each group. Rats in the BOO group exhibited increased residual urine volume, decreased voiding threshold and interval (*p* < 0.001), and notable non-voiding contractions (NVC) in the pressure curves (shown in the Fig. **4B**). The 1× HaungE group displayed an elevated micturition threshold (*p* < 0.05) and a lengthened micturition interval (*p* < 0.01). The 2× HuangE group exhibited the most significant enhancements, including a reduction in residual urine volume (*p* < 0.01) and increases in micturition threshold (*p* < 0.01) and interval (*p* < 0.001). However, the improvements in the 0.5× HuangE group did not reach statistical significance compared to the BOO group (shown in the Fig. **4C** and Table **3**).

### 3.4 Histomorphological Changes

The alterations in bladder size and morphology observed during cystostomy are shown in the Fig. (**5**). A. The bladder in the Sham group measured approximately 1.5 cm when filled. In contrast, rats in the BOO group exhibited a significantly larger bladder size of around 3.5 cm and displayed a wrinkled appearance. The bladder in the 2× HuangE group displayed sizes and morphologies that fell between the other two groups. The ratio of bladder wet weight to body weight in the BOO group was significantly higher than in the Sham group (*p* < 0.001). A decrease in this ratio was noted in the 1× and 2× HuangE groups (*p* < 0.001), while the change in the 0.5× HuangE group was not statistically significant (shown in the Fig. (**5B**) and Table **2**). The digital scanning results following Masson staining indicated a significant increase in the collagenous tissue percentage in the BOO group (*p* < 0.001). The images showed hypertrophied smooth muscle and collagenous tissue infiltrating the intermuscular bundles within the field of view. There was a reduction in the collagenous tissue percentage after HEC treatment. The 2× HuangE group displayed continuous smooth muscle tissue with a morphology resembling that of the Sham group (shown in Fig. **5C**).

### 3.5 Alterations in TNF-α, IL-6, SOD and MDA Levels

The levels of TNF-α, IL-6, SOD and MDA indicate oxidative stress and inflammation in bladder tissue. Compared to the Sham group, the expressions of TNF-α, IL-6 and MDA increased in the BOO group, while total SOD level decreased (all *p* < 0.001). In the 1× HuangE group, IL-6 (*p* < 0.001) and TNF-α (*p* < 0.05) expressions decreased compared to the BOO group. The 2× HuangE group exhibited a significant increase in total SOD level (*p* <0.01) and decreases in IL-6 (*p* <0.001), TNF-α (*p* <0.001) and MDA (*p* <0.05) levels. However, none of levels in the 0.5× groups showed statistical significance compared to the BOO group (Fig. **6**).

### 3.6 Expression of RhoA, ROCK1, and ROCK2 Proteins

Protein expression levels of the Rho-Kinase pathway were validated based on the network pharmacology results. The expression of RhoA (*p* < 0.01), ROCK1 (*p* < 0.05), and ROCK2 (*p* < 0.01) proteins in the bladder tissue in BOO group were higher than those in the Sham group. RhoA expression in the 1× HuangE group was lower than that in the BOO group (*p* < 0.05). Rats in the 2× group exhibited reduced protein expression of ROCK1 and ROCK2 compared to the BOO group (*p* < 0.05), while RhoA expression was significantly lower (*p* < 0.01). The protein expression in the 0.5× HuangE group did not differ significantly from that in the BOO group (Fig. **7**).

## 4 DISCUSSION

OAB is a complex, multifactorial symptomatic syndrome. The origin of OAB is widely accepted to be "myogenic", "neurogenic," or "urotheliogenic" [17]. BOO is commonly observed in aging males with BPH, leading to storage symptoms that might persist even after the prostatectomy [10]. BOO induces increased voiding resistance in rats, mirroring the pathophysiological processes of non-neurogenic OAB pathogenesis in human [10]. Detrusor overactivity (DO) and inflammation are the pivotal factors in this process [18, 19]. BOO triggers DO as a compensatory mechanism to enhance bladder contraction force for urine expulsion, leading to bladder smooth muscle hypertrophy, fibrosis and subsequent cyclic ischemia reperfusion injury [15]. Meanwhile, the use of urodynamics to objectively assess DO has been widely employed in studies of OAB, as the subjective symptom of urinary urgency cannot be accurately represented in animal models [17]. In this study, rats in the BOO group exhibited alteration in urination frequency, urodynamic outcomes and histomorphology, aligning with previous findings [13, 20-22]. Continuous treatment with HEC improved these lesions in rats. The enhancement of the indices became increasingly pronounced with higher drug doses, with the most significant ameliorations observed in the 2× HuangE group, indicating a dose-dependent therapeutic role of HEC on OAB management.

Network pharmacology provides insights into the potential mechanisms of complex herbal medicines through the interconnections between drug active ingredients and disease targets. Within the HEC-OAB PPI network, inflammatory factors such as IL-6 and TNF-α were prominently featured, suggesting that anti-inflammatory effects are key mechanisms for HEC treatment of OAB. GO and KEGG methods annotate and enrich the pathways associated with the respective genes and targets through their databases. The results indicate that the G protein signaling pathway coupled to muscarinic receptors may be the key pathway of action, which appears to be closely interconnected with the cAMP signaling pathway and Ca^2+^ signaling pathway. This aligns with targets such as CHRM2, CHRM3, ROCK2, and ACHE in the PPI network, linking to the main pharmacological treatment modalities for OAB. In the OAB management, antimuscarinic agents have been the first-line choice for pharmacological treatment [4]. These agents reduce compensation by antagonizing the binding of muscarinic receptors and ACHE to diastolic bladder smooth muscle. The interaction of HEC with receptors like CHRM2 and CHRM3 implies a muscarinic receptor-like antagonism that it may induce. BOO enhances the muscarinic receptor-coupled Rho/Rho-kinase pathway to increase bladder contractility [23, 24]. Excessive inflammatory response is inextricably linked to fibrosis. Persistent inflammation and oxidative stress will lead to decompensation and fibrosis, accelerating BOO progression [25]. Therefore, the reduction of bladder smooth muscle compensation and fibrosis, along with anti-inflammatory and antioxidant properties, are key mechanisms for improving OAB.

The contractile state of smooth muscle is driven by phosphorylation of regulatory myosin light chains (MLC). This process is closely related to Ca^2+^ dependent activation of RhoA and cAMP elevation leading to dis-inhibition and restoration of myosin light chain phosphatase (MLCP) activity, which suggests that the Rho/Rho-kinase pathway may be critical [26]. ROCK2 and ROCK1 are a set of isozymes, and targets for the small GTPase RhoA, which promote reorganization of the actin cytoskeleton and regulate cell shape, attachment, and motility, closely related to fibrosis [27]. They act as regulators of the actin cytoskeleton through inhibitory phosphorylation at Thr696 and Thr853 of the myosin binding subunit of MLCP, thereby leading to MLC phosphorylation and actomyosin stress fiber formation. ROCK inhibitors are thought to influence fibrotic processes through multiple pathways, including cellular mechanisms such as inflammation and oxidative stress [28]. For instance, TNF-α has been proved to upregulate ROCK2 expression in the liver *via* the NF-κB pathway [29]. The mechanisms of antimuscarinic agents involve antagonizing muscarinic receptors to decrease guanine nucleotide exchange factor (GEF) expression, leading to reduced RhoA GTPase activity and subsequent downregulation the expression of Rho-kinase pathway expression, distasting bladder smooth muscle, and reducing pronation and fibrosis [27, 30]. IL-6 and TNF-a are significant inflammatory factors and crucial targets within the PPI network of HEC-OAB, while SOD and MDA serve as markers of oxidative stress in the bladder tissue [29].

Our study demonstrates that HEC could reduce the inflammatory response and oxidative stress in bladder tissues, improving BOO-induced OAB in rats by suppressing the Rho/Rho-kinase pathway to mitigate bladder tissue fibrosis (shown in the Fig. **8**). In addition, OAB in men was considered to be always caused by BOO secondary to BPH [31]. The combination therapy for BPH, such as alpha-blockers, is recommended for OAB management in men [32]. HEC can antagonize alpha-1 receptors and inhibit prostatic epithelial proliferation, thereby suppressing prostatic hyperplasia [33]. Benefited from the advantages of multi-target therapy of TCM preparation, HEC could elicit a synergistic therapeutic effect for OAB patients with BPH in men. Additionally, early administration of HEC early in BPH may have a prophylactic effect on OAB and reduce patient burden.

Since HEC was initially developed as a treatment for BPH in males, this study was designed to investigate its extra effects on BOO-induced OAB in male rats, following existing HEC guidelines. Despite OAB having a heterogeneous presentation of symptoms between genders, there is limited evidence of gender-specific pharmacokinetic or pharmacodynamic differences in antimuscarinic drugs. The use of antimuscarinic agents as a primary treatment option is consistent between genders [34]. Though we propose that HEC is also an effective medication for OAB in women, subsequent controlled trials are necessary to figure out whether gender differences exist. Additionally, given the differences in pathogenic mechanisms between non-neurogenic and neurogenic OAB, further studies are required to evaluate the efficacy of HEC in the latter. In conclusion, we recommend HEC as a TCM treatment option for OAB in men, emphasizing the necessity for multicenter clinical studies to support our assertion. We advocate for further community-based investigations to determine the therapeutic efficacy of HEC.

## CONCLUSION

HEC improved bladder function and morphometry in BOO-induced OAB rats by reducing the expression of RhoA, ROCK1, and ROCK2 and inhibiting inflammatory reactions and oxidative stress, including fewer urination frequency, reduced residual urine volume, longer micturition intervals, higher micturition thresholds, decreased bladder weight to body weight ratio, and less collagenous tissue in the bladder tissue. We provided novel findings supporting the evidence-based use of HEC on OAB management in men within TCM practices.

## Figures and Tables

**Fig. (1):**
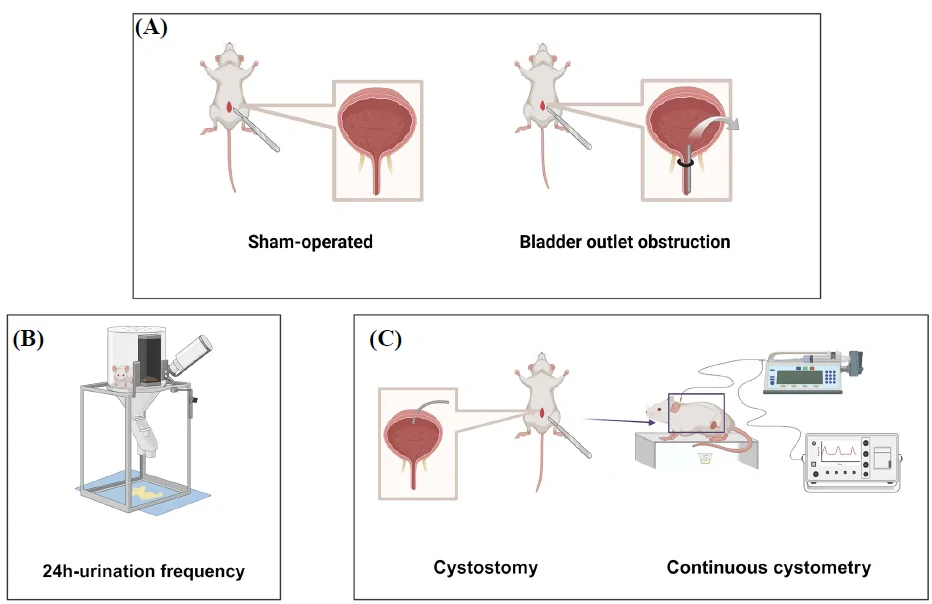
Schematic of the surgical operation and continuous cystometry. (**A**) Calibration rod was used to make a suitable ligature and removed on completion when establishing bladder outlet obstruction models. (**B**) Prepared copper sulfate supersaturated test strips were placed beneath the cages to measure the number of urinations over a 24-hour period. (**C**) Continuous cystometry was performed on day 3 post-cystostomy while the rats were awake. The rats were immobilized on a special rack for urine collection and volume measurement.

**Fig. (2):**
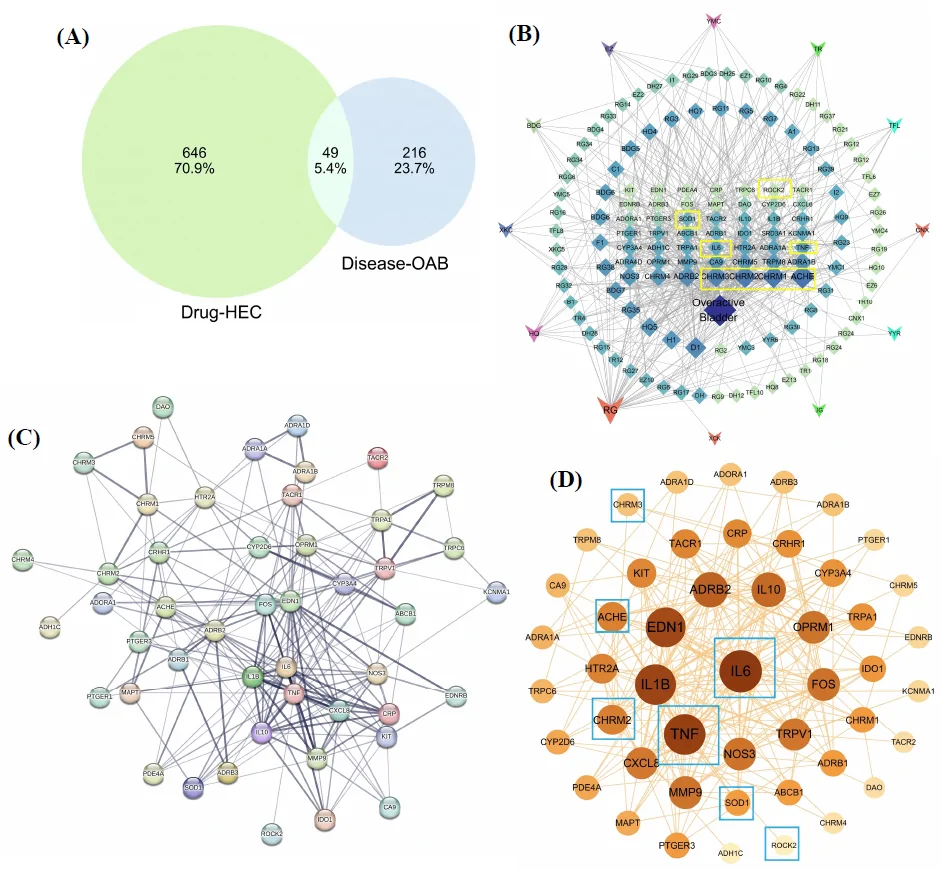
Screening of core targets between HEC and OAB. (**A**) Venn plot of HEC and OAB intersection targets. (**B**) Network plot represents “drugs-active ingredients-targets-pathways-diseases”. Considering the simplicity of the images, we used Chinese abbreviations of drugs and active ingredients, which can be found in the Supplementary-Table. **S1** for their name references. (**C**-**D**) PPI network and interaction maps. IL-6 and TNF are in the middle position, indicating their strong connection with the nodes. HEC: HuangE capsules, OAB: overactive bladder, PPI: protein-protein interaction.

**Fig. (3):**
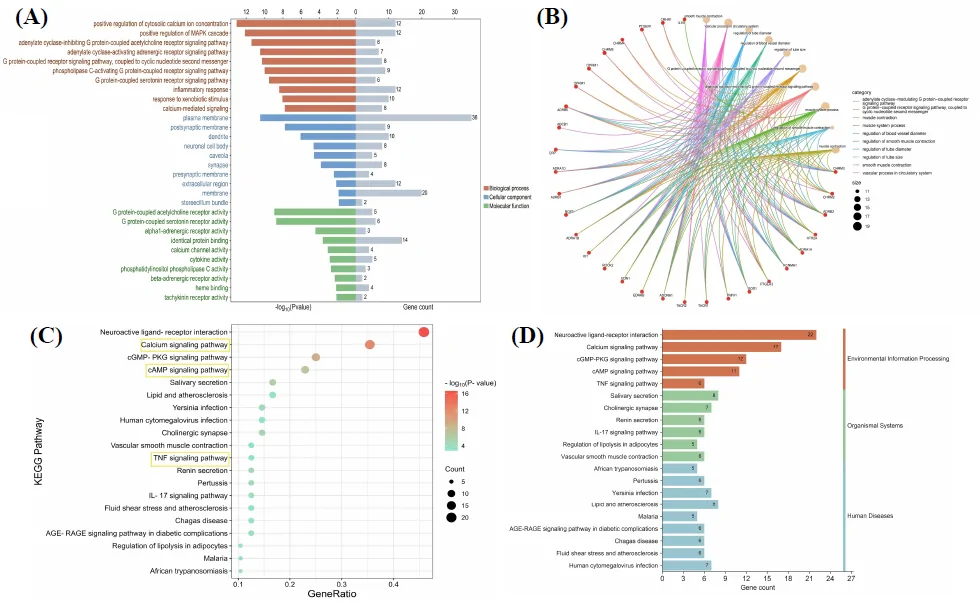
Visual plots of GO and KEGG pathway enrichment analysis. (**A**) Top 10 enriched pathways under BP, CC, and MF entries. (**B**) Cnetplot of genes associated with the enrichment pathway under BP entries. (**C**-**D**) Top 20 pathways and their classification in summary for KEGG analysis. GO: Gene Ontology, KEGG: Kyoto Encyclopedia of Genes and Genomes, BP: biological process, CC: cellular component, MF: molecular function.

**Fig. (4):**
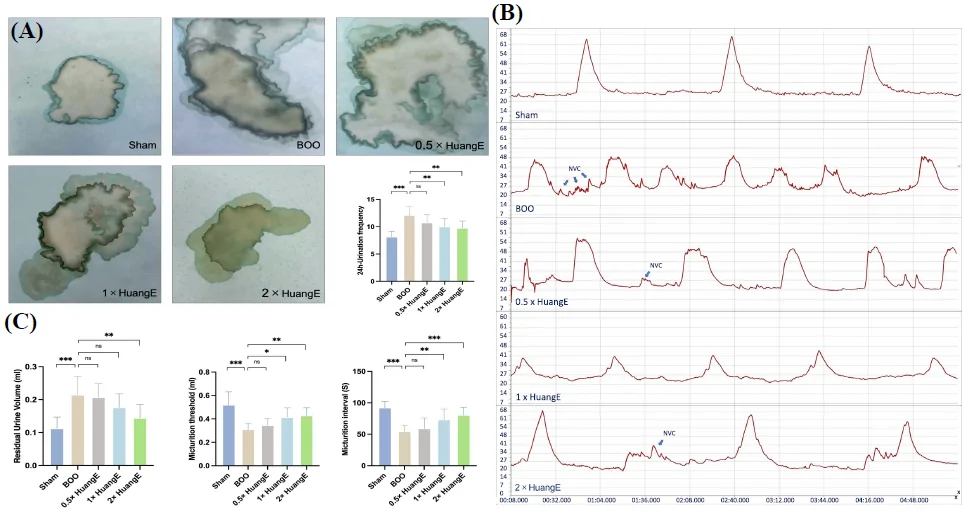
Alterations in urinary frequency and urodynamic outcomes in each group (*n=8*). (**A**) Representative test paper reflecting the frequency of urination during the same time; (**B**) Representative intravesical pressure curves; (**C**) Urodynamic findings in residual urine volume, micturition threshold, and micturition interval. **Abbreviations:** NVC: non-voiding contraction; *: *p* < 0.05, **: *p* < 0.01, ***: *p* < 0.001, ns: no significance.

**Fig. (5):**
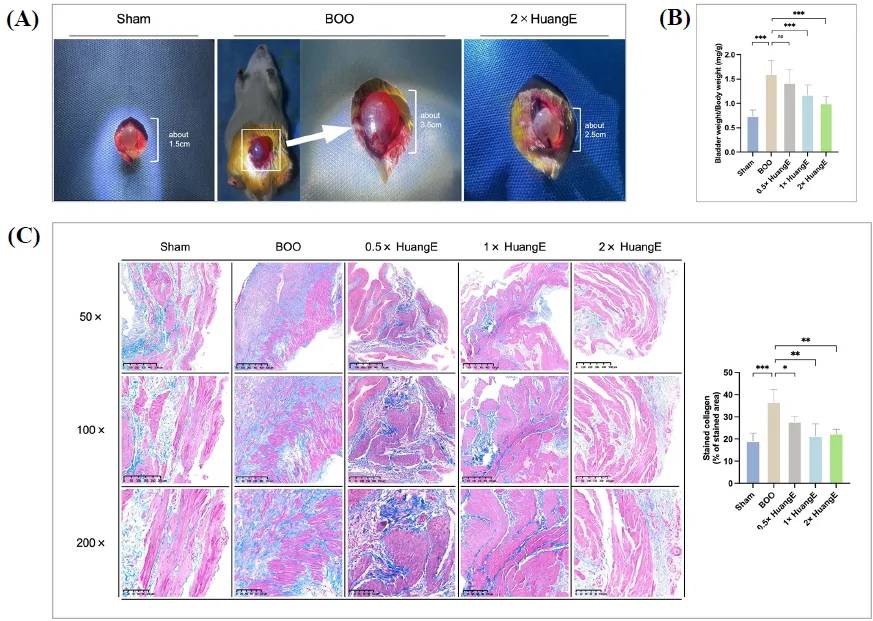
Histomorphological changes in rats' bladder tissues between different groups. (**A**) Bladder size and morphological alterations observed during cystostomy. (**B**) Histogram depicting the wet weight to body weight ratio (mg/g) of rat bladders (*n=8*). (**C**) Masson-stained image of bladder tissue under digital scanning and histogram of the percentage of collagen tissue in each group (*n=3*). Smooth muscle tissue was stained red, and collagen tissue was stained blue. *: *p* < 0.05, **: *p* < 0.01, ***: *p* < 0.001, ns: no significance.

**Fig. (6):**
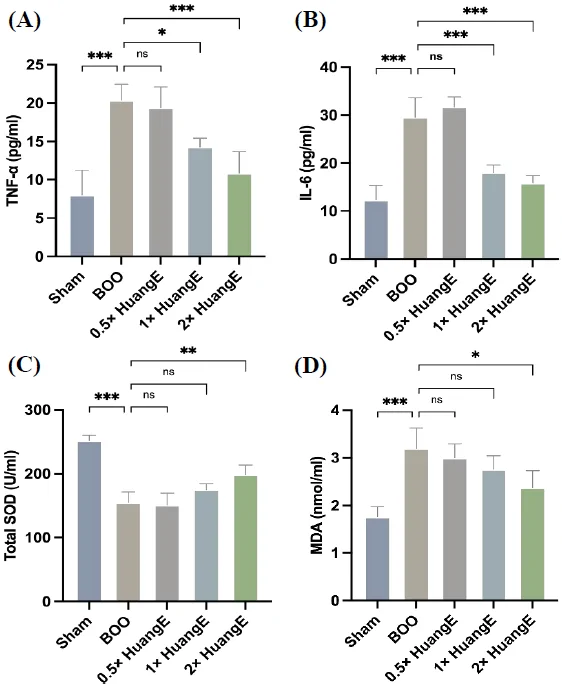
(**A-D**): The levels of TNF-α, IL-6, total SOD and MDA in bladder tissues from each group (*n*=3). TNF-α: tumor necrosis factor alpha, IL-6: interleukin 6, SOD: superoxide dismutase, MDA: malondialdehyde, *: *p* < 0.05, **: *p* < 0.01, ***: *p* < 0.001, ns: no significance.

**Fig. (7):**
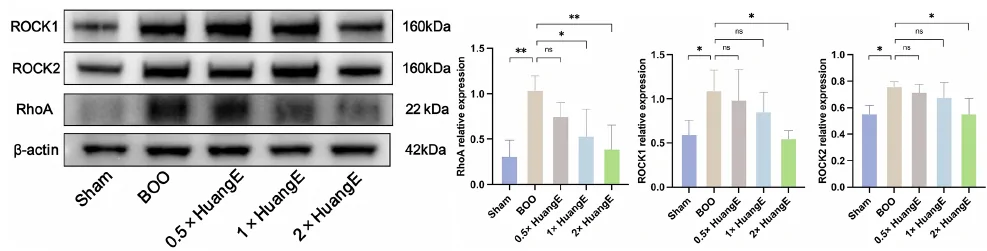
Representative western blotting images of RhoA, ROCK1, ROCK2, and quantitative analysis of the relative expression of each protein (*n*=3). *: *p* < 0.05, **: *p* < 0.01, ns: no significance.

**Fig. (8):**
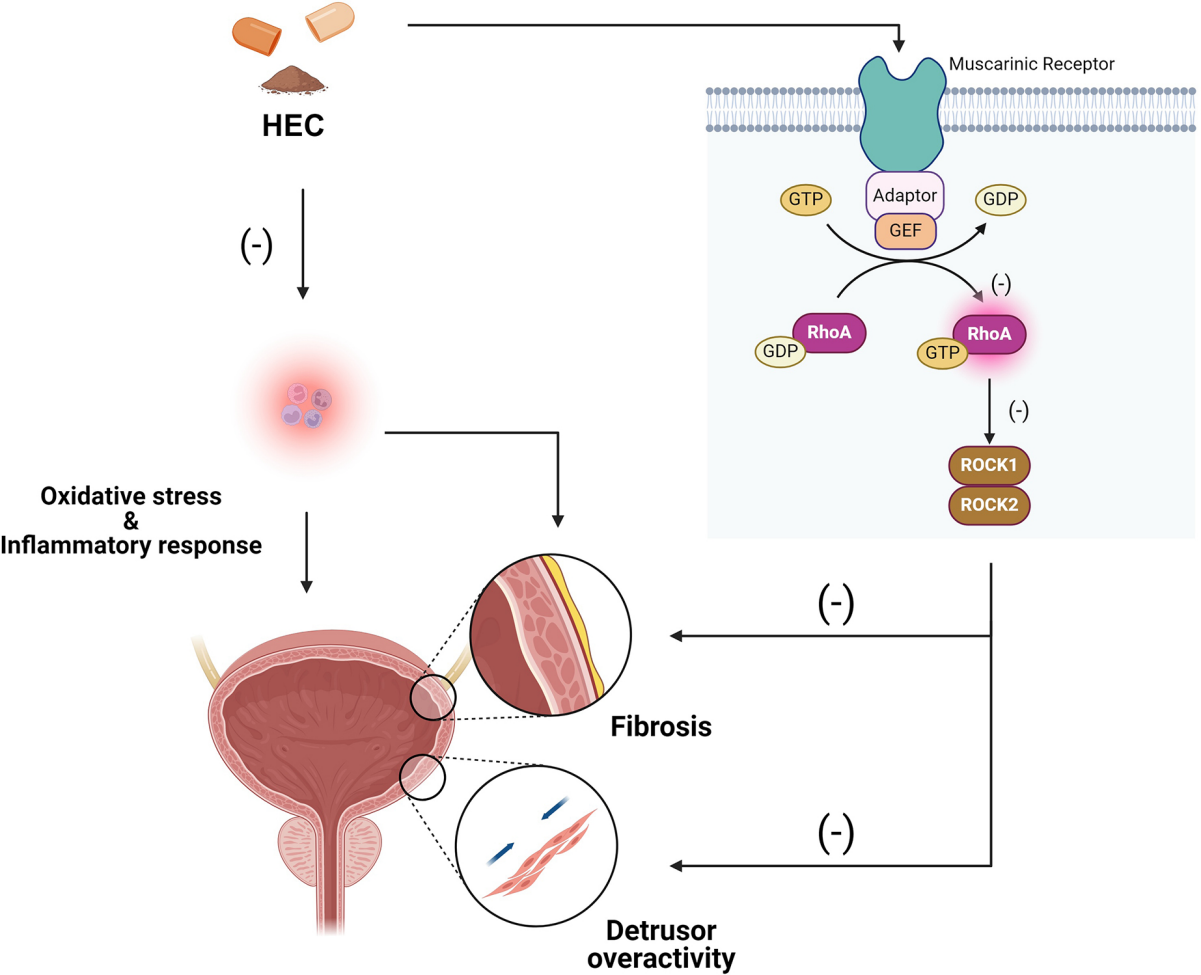
Schematic representation for the influence of HEC on the bladder.

**Table 1:** The composition of HuangE capsules. The proportion of ingredients can be found in the Chinese Pharmacopoeia Commission.

**Chinese Name**	**Latin Name**	**Proportions**
Huang qi	*Astragalus mongholicus Bunge*	11.63 %
Tao ren	*Prunus spinosa L.*	6.98 %
E zhu	*Curcuma aeruginosa Roxb.*	6.98 %
Da huang	*Rheum palmatum L.*	0.78 %
Tu fuling	*Smilax glabra Roxb.*	9.30 %
Yi yiren	*Coix lacryma-jobi L.*	15.50 %
Yi mucao	*Leonurus japonicus Houtt.*	11.63 %
Xia kucao	*Prunella vulgaris L.*	11.63 %
Rougui	*Cinnamomum cassia （L.） J. Presl*	4.65 %
Bei dougen	*Menispermum dauricum DC.*	6.98 %
Jie geng	*Platycodon grandiflorus (Jacq.) A.DC.*	6.98 %
Chuan niuxi	*Cyathula officinalis K.C. Kuan*	6.98 %

**Table 2:** The top ten potential active ingredients of HuangE capsules screening by the traditional Chinese medicine systems pharmacology database and analysis platform.

**Rank**	**Active ingredients**	**Degree**	**Sources**
1	Quercetin	20	*Astragalus mongholicus Bunge* *Leonurus japonicus Houtt.* *Cyathula officinalis K.C. Kuan* *Prunella vulgaris L.* *Smilax glabra Roxb.*
2	7-O-methylisomucronulatol	14	*Astragalus mongholicus Bunge*
3	Beta-sitosterol	14	*Cyathula officinalis K.C. Kuan* *Prunella vulgaris L.* *Prunus spinosa L.* *Rheum palmatum L.* *Smilax glabra Roxb.*
4	Cinnamyl cinnamate	13	*Cinnamomum cassia (L.) J. Presl*
5	Dibutyl Phthalate	12	*Cinnamomum cassia (L.) J. Presl*
6	l-SPD	12	*Menispermum dauricum DC.*
7	Stigmasterol	11	*Coix lacryma-jobi L.* *Prunella vulgaris L., Smilax glabra Roxb.*
8	Stepharine	11	*Menispermum dauricum DC.*
9	Cheilanthifoline	11	*Menispermum dauricum DC.*
10	3,9-di-O-methylnissolin	10	*Astragalus mongholicus Bunge*

**Table 3:** Continuous cystometry data were recorded under awake conditions (data are presented as the mean ±standard deviation). *P< 0.05, **P< 0.01 and ***P< 0.001 *vs*. the sham group; #P< 0.05, ##P< 0.01 and ###P< 0.001 *vs*. the BOO group.

	**24h-Urination Frequency**	**Bladder Weight/Body Weight (mg/g)**	**Residual Urine Volume (mL)**	**Baseline Pressure (mL)**	**Peak Pressure (mmH_2_O)**	**Micturition Threshold (mL)**	**Micturition Interval (S)**
Sham	8.13±0.99	0.74±0.12	0.11±0.03	24.82±3.16	48.55±11.76	0.52±0.11	92.46±10.01
BOO	12.16±1.55***	1.59±0.29***	0.22±0.05***	27.44±4.50	53.42±7.37	0.31±0.05***	54.40±10.20***
0.5× HuangE	10.75±1.49	1.42±0.28	0.21±0.04	26.83±4.11	54.01±9.81	0.34±0.06	59.03±17.03
1× HuangE	10.00±1.51^##^	1.17±0.21^###^	0.18±0.04	26.71±3.92	50.69±10.88	0.41±0.08^#^	73.34±16.87^##^
2× HuangE	9.75±1.28^##^	1.00±0.14^###^	0.14±0.04^##^	25.51±2.75	52.60±8.63	0.42±0.06^##^	80.66±12.57^###^

## Data Availability

The data that has been used is confidential, except those provided in the supplementary material.

## LIST OF ABBREVIATIONS

BOO: Bladder outlet obstruction
BP: Biological process
BPH: Benign prostatic hyperplasia
CC: Cellular component
CHDD: Cleaning heat and disinhibit dampness
CNMPA: China national medical products administration
DO: Detrusor overactivity
ELISA: Enzyme-linked immunosorbent assay
GEF: Guanine nucleotide exchange factor
GO: Gene ontology
HEC: HuangE capsules
IL-6: Interleukin 6
KEGG: Kyoto encyclopedia of genes and genomes
MDA: Malondialdehyde
MF: Molecular function
MLC: Myosin light chain
MLCP: Myosin light chain phosphatase
OAB: Overactive bladder
PPI: Protein–protein interaction
QoL: Quality of life
RhoA: Ras homolog gene family member A
ROCK1: Rho-associated coiled-coil-containing kinase 1
ROCK2: Rho-associated coiled-coil-containing kinase 2
SOD: Superoxide dismutase
TCM: Traditional Chinese medicine
TCMSP: Traditional Chinese medicine systems pharmacology database and analysis platform
TNF-α: Tumor necrosis factor alpha

## DECLARATION

Graphical abstract, Figure, Figures **1** and Figure. **8** were created with BioRender.com. These figures were exported under paid subscriptions.

## References

[1] Abrams P., Cardozo L., Fall M., Griffiths D., Rosier P., Ulmsten U., van Kerrebroeck P., Victor A., Wein A. (2002). The standardisation of terminology of lower urinary tract function: Report from the standardisation sub‐committee of the international continence society.. Neurourol. Urodyn..

[2] Irwin D.E., Milsom I., Kopp Z., Abrams P., Artibani W., Herschorn S. (2009). Prevalence, severity, and symptom bother of lower urinary tract symptoms among men in the EPIC study: Impact of overactive bladder.. Eur. Urol..

[3] Cheng Y., Chen T., Zheng G., Song Z., Zhang G., Rao X., Zeng T., Yuan C. (2024). Prevalence and trends in overactive bladder among men in the United States, 2005–2020.. Sci. Rep..

[4] De Nunzio C., Brucker B., Bschleipfer T., Cornu J.N., Drake M.J., Fusco F., Gravas S., Oelke M., Peyronnet B., Tutolo M., van Koeveringe G., Madersbacher S. (2021). Beyond antimuscarinics: A review of pharmacological and interventional options for overactive bladder management in men.. Eur. Urol..

[5] Zhang L., Shen J., Zeng F., Shao K., Li Q., Li D., Li Y., Gao Z. (2019). A review of the combined diagnosis and treatment of overactive bladder syndrome in Chinese and Western medicine.. World J. Integr. Tradit. Western Med..

[6] Li H. (2021). Expert consensus on the integration of Chinese and Western medicine in the pharmacological treatment of benign prostatic hyperplasia.. Chin. J. Androl..

[7] Yue P., Zhixian Y. (2024). Therapeutical effect of Huang’e capsule on bladder dysfunction after radical prostatectomy for prostatecancer.. Shandong Yiyao.

[8] Sherman B.T., Hao M., Qiu J., Jiao X., Baseler M.W., Lane H.C., Imamichi T., Chang W. (2022). DAVID: A web server for functional enrichment analysis and functional annotation of gene lists (2021 update).. Nucleic Acids Res..

[9] Tang D., Chen M., Huang X., Zhang G., Zeng L., Zhang G., Wu S., Wang Y. (2023). SRplot: A free online platform for data visualization and graphing.. PLoS One.

[10] Shen J.D., Chen S.J., Chen H.Y., Chiu K.Y., Chen Y.H., Chen W.C. (2021). Review of animal models to study urinary bladder function.. Biology.

[11] Limprasutr V., Sharp P., Jampachaisri K., Pacharinsak C., Durongphongtorn S. (2021). Tiletamine/zolazepam and dexmedetomidine with tramadol provide effective general anesthesia in rats.. Animal Model. Exp. Med..

[12] Wei W., Wu X., Li Y. (2010). Methodology of Pharmacological Experiment.

[13] Yu Q., Wu C., Chen Y., Li B., Wang R., Huang R., Li X., Gu D., Wang X., Duan X., Li S., Liu Y., Wu W., Hennenberg M., Zeng G. (2021). Inhibition of LIM kinase reduces contraction and proliferation in bladder smooth muscle.. Acta Pharm. Sin. B.

[14] Wang J., Chen Y., Gu D., Zhang G., Chen J., Zhao J., Wu P. (2017). Ketamine-induced bladder fibrosis involves epithelial-to-mesenchymal transition mediated by transforming growth factor-β1.. Am. J. Physiol. Renal Physiol..

[15] Majima T., Matsukawa Y., Funahashi Y., Kato M., Yamamoto T., Gotoh M. (2020). The effect of mirabegron on bladder blood flow in a rat model of bladder outlet obstruction.. World J. Urol..

[16] Kanehisa M., Furumichi M., Sato Y., Kawashima M., Ishiguro-Watanabe M. (2023). KEGG for taxonomy-based analysis of pathways and genomes.. Nucleic Acids Res..

[17] Peyronnet B., Mironska E., Chapple C., Cardozo L., Oelke M., Dmochowski R., Amarenco G., Gamé X., Kirby R., Van Der Aa F., Cornu J.N. (2019). A comprehensive review of overactive bladder pathophysiology: On the way to tailored treatment.. Eur. Urol..

[18] Nocchi L., Daly D.M., Chapple C., Grundy D. (2014). Induction of oxidative stress causes functional alterations in mouse urothelium via a TRPM 8‐mediated mechanism: Implications for aging.. Aging Cell.

[19] Bouchelouche K., Alvarez S., Horn T., Nordling J., Bouchelouche P. (2006). Human detrusor smooth muscle cells release interleukin-6, interleukin-8, and RANTES in response to proinflammatory cytokines interleukin-1β and tumor necrosis factor-α.. Urology.

[20] Nageib M., Zahran M.H., El-Hefnawy A.S., Barakat N., Awadalla A., Aamer H.G., Khater S., Shokeir A.A. (2020). Low energy shock wave‐delivered intravesical botulinum neurotoxin‐A potentiates antioxidant genes and inhibits proinflammatory cytokines in rat model of overactive bladder.. Neurourol. Urodyn..

[21] Choi J.B., Jeon S.H., Kwon E.B., Bae W.J., Cho H.J., Ha U.S., Hong S.H., Lee J.Y., Kim S.W. (2020). The effects of oral administration of the novel muscarinic receptor antagonist DA-8010 on overactive bladder in rat with bladder outlet obstruction.. BMC Urol..

[22] Wishahi M., Hassan S., Kamal N., Badawy M., Hafiz E. (2024). Is bladder outlet obstruction rat model to induce overactive bladder (OAB) has similarity to human OAB? Research on the events in smooth muscle, collagen, interstitial cell and telocyte distribution.. BMC Res. Notes.

[23] Andersson K.E. (2011). Antimuscarinic mechanisms and the overactive detrusor: An update.. Eur. Urol..

[24] Takahashi N., Shiomi H., Kushida N., Liu F., Ishibashi K., Yanagida T., Shishido K., Aikawa K., Yamaguchi O. (2009). Obstruction alters muscarinic receptor‐coupled RhoA/Rho‐kinase pathway in the urinary bladder of the rat.. Neurourol. Urodyn..

[25] Metcalfe P.D., Wang J., Jiao H., Huang Y., Hori K., Moore R.B., Tredget E.E. (2010). Bladder outlet obstruction: Progression from inflammation to fibrosis.. BJU Int..

[26] Khromov A., Choudhury N., Stevenson A.S., Somlyo A.V., Eto M. (2009). Phosphorylation-dependent autoinhibition of myosin light chain phosphatase accounts for Ca2+ sensitization force of smooth muscle contraction.. J. Biol. Chem..

[27] El Masri R., Delon J. (2021). RHO GTPases: From new partners to complex immune syndromes.. Nat. Rev. Immunol..

[28] Yu B., Sladojevic N., Blair J.E., Liao J.K. (2020). Targeting Rho-associated coiled-coil forming protein kinase (ROCK) in cardiovascular fibrosis and stiffening.. Expert Opin. Ther. Targets.

[29] Alexandre E.C., Calmasini F.B., Sponton A.C.S., de Oliveira M.G., André D.M., Silva F.H., Delbin M.A., Mónica F.Z., Antunes E. (2018). Influence of the periprostatic adipose tissue in obesity-associated mouse urethral dysfunction and oxidative stress: Effect of resveratrol treatment.. Eur. J. Pharmacol..

[30] Braverman A.S., Doumanian L.R., Ruggieri M.R. (2006). M2 and M3 muscarinic receptor activation of urinary bladder contractile signal transduction. II. Denervated rat bladder.. J. Pharmacol. Exp. Ther..

[31] Wagg A. (2021). Male overactive bladder: Underappreciated, under-researched. more please?. Eur. Urol..

[32] Yanagida K., Watanabe D., Yoshida T., Mizushima A., Nakagawa T. (2024). The effects of vibegron add-on therapy on Alpha 1-blocker therapy for sexual function and overactive bladder symptoms in benign prostatic hyperplasia: A prospective, open-label study.. J. Clin. Med..

[33] Ru-wei W., Jian-feng Y., Shen L., Jin-ming J., Ke-qin N., Lei C., Shu-shuo L. (2012). Analysis on efficacy of Huang’e Capsule on benign prostatic hyperplasia.. Chin. Tradit. Herbal Drugs.

[34] Hartigan S.M., Dmochowski R.R. (2020). Gender specific pharmacokinetic and pharmacodynamic considerations for antimuscarinic drugs for overactive bladder treatment.. Expert Opin. Drug Metab. Toxicol..

